# Does Academic Productivity Correlate With Industry Payments in Hand Surgery Fellowship Programs in the United States?

**DOI:** 10.7759/cureus.47369

**Published:** 2023-10-20

**Authors:** Alexander N Khouri, Kempland C Walley, Anthony N Baumann, Albert T Anastasio, Seung-Ho Bae, David Ruch

**Affiliations:** 1 Plastic Surgery, University of Michigan, Ann Arbor, USA; 2 Orthopedic Surgery, University of Michigan, Ann Arbor, USA; 3 Rehabilitation Services, University Hospitals, Cleveland, USA; 4 Orthopedic Surgery, Duke University, Durham, USA

**Keywords:** research productivity, academics, hand surgery fellowship, industry funding, hand surgery

## Abstract

Background: Hand surgeons and the industry share a common goal to improve the care of patients. However, industry support remains controversial, and the current relationship with fellowship programs remains unclear. This study explores the relationship between industry support and research productivity among hand surgeons in the academic setting.

Methods: The Open Payments database, created by the Centers for Medicare and Medicaid Services (CMS) as a result of the Sunshine Act, was used to identify supplemental income paid to physicians of hand surgery fellowships in the United States. Both lifetime individual physician and aggregated fellowship Sunshine Act supplemental income (2015-2021) were collected for review. Supplemental income only reflects royalties, consulting fees, or food and does not include direct research funding. H-index was collected through the Scopus website as a proxy for academic productivity.

Results: Six hundred and thirty-four faculty physicians (90.8%) from 94 hand surgery fellowships (100%) were included in the study. The mean individual physician lifetime supplemental income was $67,272 (median $341,861), whereas the mean individual physician H-index was 12.5 (median 9.0). There was a significant and weak positive correlation between individual physician H-index and lifetime income (p<0.001). Similarly, there was a significant and moderately positive correlation between the combined fellowship H-index and total lifetime income (p<0.001).

Conclusion: Research productivity of an orthopedic hand fellowship group and individual academic hand surgeon correlate with overall industry support from indirect research funding. Further work is required to better understand the advantages and disadvantages of industry support of academically productive hand surgeons at hand surgery fellowships.

## Introduction

Hand surgeons and the commercial industry share a common goal to improve the care of patients. This synergistic collaboration has facilitated significant advances in the field of hand surgery [1]. However, the relationship between hand surgery and industry is complex [2]. Proper oversight and disclosure of relevant conflicts of interest is necessary to prevent undue researcher bias [3]. Further, financial incentives have the potential to corrupt study results [4,5]. Due to these concerns, there remains variable involvement between academic hand surgeons and industry.

To increase transparency related to industry funding, the Physician Payments Sunshine Act was established in 2010 as part of the Affordable Care Act [6]. This resulted in the creation of the “Open Payments” database, a publicly accessible electronic record of physician financial interactions with industry [6]. The database records all payments from industry to physicians greater than $10 and records them as either general payments, ownership interests, or research payments [7]. This resource has been used across a variety of orthopedic specialties for research purposes, including hand surgery [3,8-10]. However, despite this widely available resource, the relationship between industry support and academic productivity with respect to hand surgery remains unknown.

We aimed to explore the relationship between academic productivity and industry involvement across hand surgery fellowship programs. We hypothesize that higher industry compensation, as recorded in the Open Payments Public Database, would be associated with increased total research productivity at a given fellowship. Secondarily, we assessed the relationship between the H-index of an individual surgeon and their net industry earnings. We hypothesize that higher H-index scores would be associated with increased net industry earnings.

## Materials and methods

Study search information

For industry payment information, our current study used the Centers for Medicare and Medicaid Services (CMS) website (https://openpaymentsdata.cms.gov/search) for physician lifetime earnings (dollars) and lifetime payments. For a measure of academic productivity, the Scopus website (https://openpaymentsdata.cms.gov/search) was used to determine each individual physician’s H-index. The American Society for Surgery of the Hand (ASSH) for fellowships (https://www.assh.org/applications/s/fellowshipdirectory) was used for information on the different hand orthopedic surgery fellowships in the United States. Based on the fellowships found on the ASSH website, each individual fellowship site was utilized for relevant faculty and fellow information. This study was completed in April 2023 and reflects the most up-to-date information on hand orthopedic surgery fellowships as of April 2023. 

Data extraction

Data extraction was performed by multiple authors in April 2023. Data collected in the current study included H-index per faculty physician, total lifetime earnings per faculty physician (dollars), total payments made to faculty physician, combined H-index per hand orthopedic surgery fellowship, combined lifetime earnings per hand orthopedic surgery fellowship (dollars), and average lifetime earnings per physician per hand orthopedic surgery fellowship (dollars). For subgroup analysis in the current study, individual physicians were placed in three groups depending on their individual H-index to better examine the relationship between the H-index and total lifetime earnings. The “less than 10 H-index” group included individual physicians with less than an H-index of 10. The “10-20 H-index” group included individual physicians with an H-index between 10 and 20. The “greater than 20 H-index” included individual physicians with an H-index higher than 20. 

Study definitions

For this study, total lifetime earnings are defined as the total amount of money (dollars) paid to physicians as reported on the CMS website. These earnings reflect compensation for food, consulting fees, royalties, and other industry-related payments, as seen in the category of “General Payments” on the CMS website. The category of total lifetime payments does not include research funding, which forms a separate category on the CMS website. Total lifetime payments refer to the number of times a payment was given to a physician and are recorded on the CMS website under “General Payments.”

Statistical analysis

Statistical analysis was completed using SPSS version 29.0 (IBM Corp., Armonk, NY) as the statistical software for this study. The normality of the data was tested using the Kolmogorov-Smirnov test or the Shapiro-Wilk test depending on the sample size. Three or more groups were compared using the ANOVA or Kruskal-Wallis test with Bonferroni correction to allow for better analysis of the relationships depending on the normality of the data. Significance values were set at 0.05. Correlation between variables was performed for continuous data using Pearson’s r or Spearman’s rho, depending on the normality of the data. Measures of central tendency were used for demographics to describe the data included in the current study. 

## Results

Initial search

Based on fellowship information on the HAND website, there are a total of 94 hand orthopedic surgery fellowships in the United States. Within the 94 hand orthopedic surgery fellowships, there are a total of 698 faculty physicians from which 634 physicians (90.8%) had complete data for H-index and earnings and payments from the CMS website. There were 35 physicians missing earnings and payment information from CMS and 62 physicians missing an H-index on Scopus, resulting in 64 physicians (9.2%) being excluded due to incomplete information. None of the missing data resulted in any of the hand orthopedic surgery fellowships being excluded from the study and data analysis.

Individual physician demographics

Mean individual physician (n=634 faculty physicians) H-index as reported on Scopus is 12.5 ± 12.0 (median 9.0; range, 0-90.0). Mean individual physician (n=634 faculty physicians) lifetime earnings reported on the CMS website were $67,272.64 ± $341,861.54 (median, $341,861.54; range, $9.48-$6,780,838.18). Mean individual physician (n=634 faculty physicians) lifetime payments on the CMS website were 60.4 ± 91.8 (median 27.0; range, 0-811). See Table 1 below for the H-index, total lifetime earnings, and total lifetime payments for the top five faculty physicians at hand orthopedic surgery fellowships in the United States in terms of total lifetime earnings.

**Table 1:** Study information on faculty physicians at hand orthopedic surgery fellowships in the United States with the top five total lifetime earnings (dollars). Information includes individual H-index, total lifetime earnings, and total lifetime payments paid to the individual faculty physicians.

Physician ranking by total lifetime earnings	H-index	Total lifetime earnings (dollars)	Total lifetime payments
1	48	$6,780,847.66	258
2	28	$3,284,509.15	811
3	64	$2,305,302.34	223
4	0	$1,638,986.89	77
5	12	$1,335,279.07	597

Fellowship demographics

Mean combined physician H-index per hand orthopedic surgery fellowship (n=94 fellowships) was 84.4 ± 59.7 (median 68.5; range: 5.0-307.0). Mean combined physician lifetime earnings per hand orthopedic surgery fellowship was $453,732.48 ± $934,084.73 (median: $125,595.30; range: $127.44-$6,783,053.99). See Table 2 below for the combined H-index, combined total lifetime earnings, combined total lifetime payments, and lifetime earnings per physician per fellowship program for hand orthopedic surgery fellowship programs in the United States. 

**Table 2:** Information on hand orthopedic surgery fellowships in the United States. Information includes combined H-index per fellowship, combined lifetime earnings for all physician faculty per fellowship, combined total lifetime payments, and average total lifetime earnings per faculty physician per fellowship.

Fellowship ranking by combined total lifetime earnings	Combined fellowship H-index	Combined fellowship lifetime earnings (dollars)	Combined fellowship payments	Average total lifetime earnings per fellowship per physician (dollars)
1	74	$6,783,053.99	268	$2,261,018.00
2	162	$3,739,216.03	2158	$249,281.07
3	28	$3,284,509.15	811	$3,284,509.15
4	165	$2,366,646.82	1728	$182,049.76
5	307	$1,854,249.48	1464	$154,520.79
6	127	$1,802,858.91	1168	$225,357.36
7	25	$1,723,836.37	532	$287,306.06
8	162	$1,502,545.96	651	$166,949.55
9	154	$1,486,677.70	617	$212,382.53
10	111	$1,467,495.53	949	$183,436.94
11	189	$1,190,627.03	2036	$70,036.88
12	187	$840,555.45	602	$84,055.55
13	134	$797,053.36	169	$132,842.23
14	85	$777,968.58	390	$155,593.72
15	101	$652,172.90	586	$81,521.61
16	30	$651,206.56	1269	$162,801.64
17	98	$609,993.97	1041	$60,999.40
18	240	$584,368.11	849	$53,124.37
19	49	$526,071.34	341	$131,517.84
20	163	$496,099.24	855	$62,012.41
21	72	$486,149.61	920	$121,537.40
22	159	$484,509.29	640	$60,563.66
23	112	$445,172.29	421	$74,195.38
24	120	$416,061.19	463	$83,212.24
25	105	$397,045.00	486	$44,116.11
26	56	$358,319.42	336	$71,663.88
27	128	$352,391.95	370	$39,154.66
28	78	$343,851.02	415	$42,981.38
29	78	$306,296.00	156	$61,259.20
30	40	$292,619.63	292	$41,802.80
31	24	$277,128.06	293	$92,376.02
32	20	$260,367.51	546	$52,073.50
33	209	$258,082.38	558	$28,675.82
34	102	$226,848.36	372	$18,904.03
35	68	$223,611.12	502	$27,951.39
36	147	$222,157.30	277	$37,026.22
37	154	$210,231.29	340	$19,111.94
38	40	$199,570.65	563	$33,261.78
39	63	$183,768.97	349	$26,252.71
40	200	$175,888.35	129	$25,126.91
41	84	$172,664.27	455	$17,266.43
42	179	$160,392.14	749	$8,441.69
43	48	$146,368.83	195	$29,273.77
44	158	$136,412.38	430	$22,735.40
45	49	$127,807.30	171	$25,561.46
46	98	$126,688.48	91	$31,672.12
47	38	$125,851.93	302	$20,975.32
48	176	$125,338.66	363	$11,394.42
49	29	$108,072.04	212	$27,018.01
50	73	$106,649.06	302	$9,695.37
51	44	$106,419.96	252	$21,283.99
52	63	$99,589.97	316	$16,598.33
53	63	$99,589.97	316	$16,598.33
54	24	$98,093.97	430	$14,013.42
55	25	$97,372.36	162	$19,474.47
56	106	$91,132.09	465	$11,391.51
57	142	$88,832.10	146	$12,690.30
58	67	$83,210.41	74	$16,642.08
59	54	$82,059.19	153	$13,676.53
60	40	$78,122.97	232	$13,020.50
61	120	$75,571.98	205	$6,297.67
62	173	$74,918.66	266	$5,762.97
63	32	$67,244.33	144	$22,414.78
64	57	$67,217.32	229	$11,202.89
65	55	$64,925.86	308	$10,820.98
66	46	$55,868.40	297	$6,207.60
67	69	$54,927.23	73	$6,865.90
68	38	$50,205.10	199	$12,551.28
69	18	$49,526.82	696	$6,190.85
70	36	$47,611.10	102	$11,902.78
71	28	$44,585.96	342	$8,917.19
72	70	$42,598.45	176	$6,085.49
73	120	$41,655.10	111	$8,331.02
74	77	$38,897.56	418	$4,862.20
75	123	$33,857.56	214	$4,232.20
76	109	$32,140.89	125	$5,356.82
77	27	$31,580.20	190	$7,895.05
78	23	$30,677.20	179	$30,677.20
79	42	$29,100.36	64	$7,275.09
80	5	$26,438.46	167	$13,219.23
81	48	$24,842.49	64	$4,140.42
82	90	$19,305.40	105	$2,145.04
83	70	$19,017.14	52	$3,803.43
84	45	$17,758.04	54	$5,919.35
85	28	$15,452.09	196	$5,150.70
86	47	$15,086.39	139	$1,508.64
87	32	$13,959.08	106	$2,791.82
88	51	$13,651.25	85	$2,275.21
89	40	$13,188.64	67	$3,297.16
90	27	$7,324.24	39	$1,831.06
91	14	$6,259.95	27	$2,086.65
92	15	$4,748.83	97	$1,187.21
93	16	$2,640.07	14	$2,640.07
94	20	$127.44	2	$127.44

Lifetime earnings and H-index relationships for individual physicians

**Figure 1:**
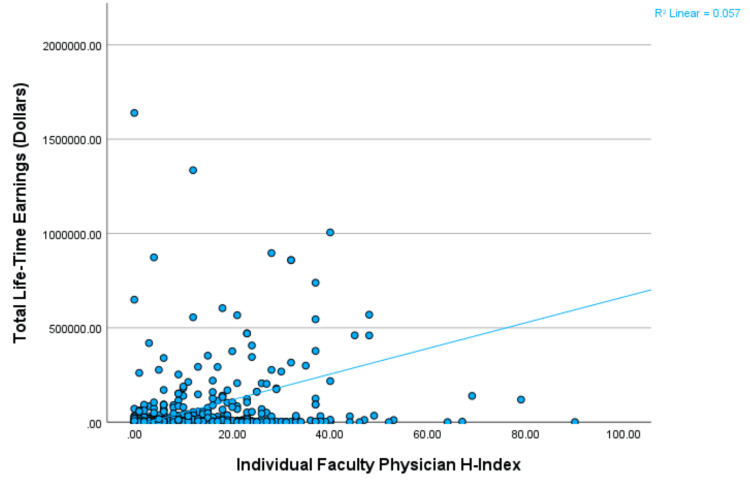
Scatter plot of earnings by H-index. Significant but weak positive correlation between individual faculty H-index and individual physician lifetime earnings associated with hand orthopedic surgery fellowships in the United States. The line represents the best-fit line for correlation. The Y-axis was fitted to $2,000,000 to exclude several outliers for better visualization of the relationship between the H-index and the total lifetime earnings of individual physicians at hand orthopedic fellowships.

There was a significant and weak positive correlation between individual physician H-index and individual physician lifetime earnings (p<0.001; Spearman’s rho=0.141). See Figure 1 for the scatter plot with a best-fit line showing a correlation between the H-index of individual faculty physicians at hand orthopedic surgery fellowships and total lifetime earnings. There was a significant difference between individual faculty H-index (“less than 10 H-index”, “10-20 H-index”, and “greater than 20 H-index”) and individual faculty lifetime earnings (Kruskal-Wallis H=9.431; p=0.009). The “greater than 20 H-index” group (n=127 faculty physicians) had significantly more total lifetime earnings than the “less than 10 H-index” group (n=327 physicians) (p=0.008) but not compared to the “10-20 H-index” group (n=180 faculty physicians) (p=0.422). The “10-20 H-index” group (n=180 faculty physicians) had no significant difference in total lifetime earnings as compared to the “less than 10 H-index” group (n=327 faculty physicians) (p=0.366). The “less than 10 H-index” group had mean total lifetime earnings of $27,113.28 ± $115,772.73 (median $4,675.10). The “10-20 H-index” group had mean total lifetime earnings of $44,491.05 ± $128,980.14 (median $5,122.57). The “greater than 20 H-index” group had mean total lifetime earnings of $202,963.95 ± $710.867.06 (median $8,155.72).

Lifetime earnings and H-index correlations for fellowship programs

**Figure 2:**
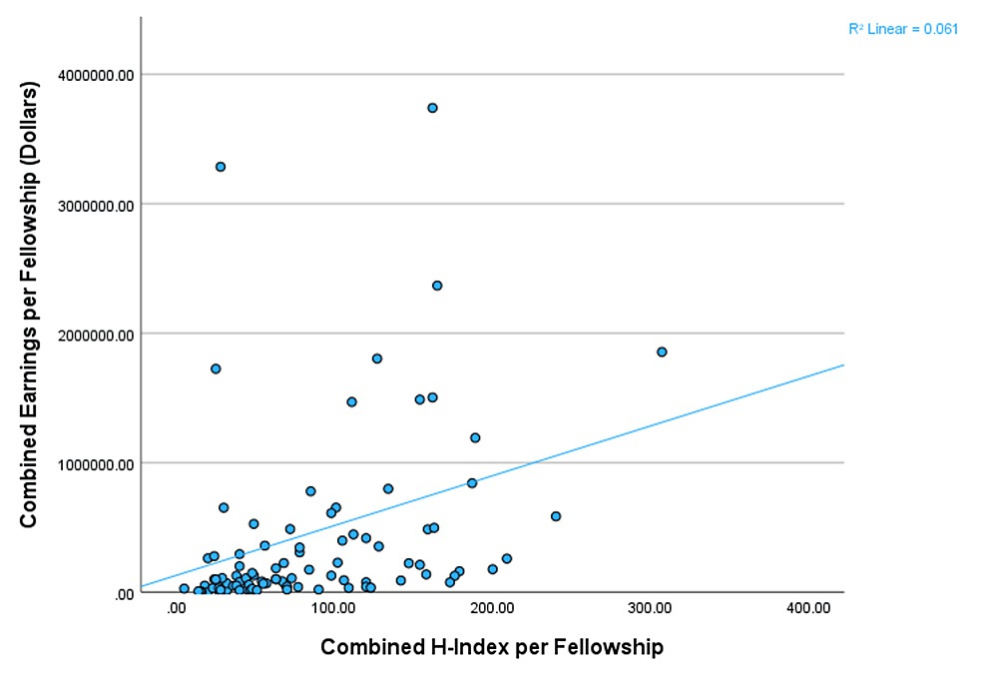
Scatter plot of earnings by H-index per fellowship. Significant and moderately positive correlation between the combined H-index per hand orthopedic surgery fellowship and the combined total lifetime earnings reported on the CMS website. The line represents the best-fit line for the correlation. The Y-axis was set to $4,000,000 for a better representation of the relationship between the combined H-index and earnings per fellowship.

There was also a significant and moderately positive correlation between the combined fellowship H-index and total lifetime earnings per fellowship (p<0.001, Spearman’s rho=0.524). See Figure 2 for a scatter plot with the best-fit line showing the significant and moderately positive correlation between the combined fellowship H-index and lifetime earnings per hand orthopedic surgery fellowship. 

## Discussion

This study explores the relationship between industry support and research productivity among hand surgeons in the academic setting, both for the entire fellowship and individual physicians. With respect to our primary hypothesis, we found a statistically significant and moderately positive correlation between the combined fellowship H-index and total lifetime earnings. Secondarily, we found a statistically significant but weak correlation between individual physician H-index and individual physician total lifetime earnings. In all, these results indicate that the research productivity of an orthopedic hand fellowship group and individual academic hand surgeons correlates with overall industry involvement. 

Payments by industry directly for research support will improve academic productivity [11]. However, this study excludes financial support explicitly for research and instead focuses on payments for other reasons such as food and drink, travel, cash payments, and cash-equivalent payments. These indirect payments were also found to correlate with H-index, both at the program-wide and individual physician levels. Several explanations are possible. Surgeons with otherwise busy clinical practices devote time and energy to explore academic passions, often without commiserate financial recompense [12]. Lack of time and revenue are commonly cited concerns among surgeons seeking academic promotion and striving for scientific progress [13]. Commercial industry support may provide physician-scientists the financial freedom to pursue academic interests by reimbursing the time and opportunity cost of pursuing research instead of expanding a clinical practice. Additionally, dissemination of research through symposia, presentations, and journal publications results in significant exposure for physicians. A limited number of academically productive and well-respected physician-scientists who frequently contribute to panels and give podium presentations at national meetings may be more commonly approached by industry for representation [14]. This financial support by industry for principal investigators at fellowship programs, in turn, can further promote fellow research endeavors and educational objectives. For these reasons, industry financial support of academically productive surgeons drives scientific progress at hand surgery fellowships.

However, there are some potential pitfalls for industry financial support of costs indirectly related to research. Surgeons with industry support may be incentivized to publish a higher total number of manuscripts rather than pursue long-term passions with the largest clinical impact. Bibliometric indices are often used by industry to measure productivity, in addition to being a predictor of promotion in academic practice [15]. This results in a large number of publications without significant scientific progress. Principal investigators may also be dissuaded by industry from submitting publications with negative outcomes or ineffective products, limiting the distribution of information and risking colleagues repeating unnecessary work [16]. Lastly, educators in hand surgery fellowships must be aware of the impact their relationship with the industry has on trainees. Fellowship training heavily influences future academic engagement, subspeciality society participation, and involvement with industry as a consultant, design-team member, or paid educator [17]. The experience faculty educators provide fellows to industry will significantly impact their involvement in the future.

In all, further research is required to better understand how financial support from industry can support the advancement of hand surgery without generating undue incentives for academic hand surgeons. However, if ultimately both the surgeon and industry firm “maintain the responsibility to the patient as paramount,” as written in the American Society for Surgery of the Hand (ASSH) code of ethics, then the synergistic relationship can propel innovation in hand surgery [18].

This study has several limitations. With any database study, there is limited access to specific fellowships or individual identifiers, which limits the gathering of data and the inclusion of programs. Second, we employ the H-index as a marker for academic productivity. While the H-index correlates with the number of publications and citations, this metric is criticized for being overly simplistic and failing to capture the actual impact of scientific investigation [19]. Further, using Scopus as a database for H-index may underestimate research productivity. Finally, we did not control for the age of a given surgeon or the average age of the surgeons at a given fellowship because this information was not available in the database. The H-index is associated with age and industry involvement may correlate with years of experience within hand surgery, rather than academic productivity alone [20]. Controlling for age, time in practice, or adding a control group of academic hand surgeons who do not receive industry support would strengthen these findings and will be incorporated in future studies. Last, these results are applicable to the academic environment and may not translate to relationships with industry within private or privademic settings.

## Conclusions

In conclusion, our study found a statistically significant correlation between individual physician H-index and total lifetime, non-research-related earnings as reported on CMS. This finding remained true and was further strengthened when H-index and total lifetime earnings were combined for the entire faculty at a given orthopedic hand surgery fellowship. Further research is required to better understand the advantages and disadvantages of industry support of academically productive hand surgeons at hand surgery fellowships.
